# Novel strategies for PEDV to interfere with host antiviral immunity through Caspase-1

**DOI:** 10.1080/21505594.2025.2560890

**Published:** 2025-09-13

**Authors:** Wen Shi, Weilv Xu, Qian Lv, Zi’an Zhang, Xinyu Fu, Danyue Li, Suhui He, Yumeng Wang, Jinxia Xu, Shiyang Liu, Yuanxiang Ge, Peide Li, Changbo Ou, Xiaoliang Li, Fushan Shi

**Affiliations:** a Department of Veterinary Medicine, College of Animal Sciences, Zhejiang University, Hangzhou, China; b College of Animal Science and Technology, Guangxi University, Nanning, China; c Guangxi Key Laboratory of Animal Reproduction Breeding and Disease Control, College of Animal Science and Technology, Guangxi University, Nanning, China; d Institute of Animal Husbandry and Veterinary, Wenzhou Academy of Agricultural Sciences, Zhejiang University-Xinchang Joint Innovation Centre (TianMu Laboratory), Wenzhou, China; e Department of Animal Science, Wenzhou Vocational College of Science & Technology, Wenzhou, China; f Wenzhou Academy of Agricultural Sciences, Wenzhou, China

**Keywords:** Caspase-1, MAVS, PEDV, IFN-β

## Abstract

Porcine epidemic diarrhea virus (PEDV), a member of the *Coronaviridae* family, responsible for substantial morbidity and mortality in neonatal piglets, representing an ongoing threat to the swine industry. The type I interferon (IFN) response is integral to the innate immune system, playing a critical role in host defense against viral infection. However, viruses have evolved diverse strategies to evade or suppress host immune responses to facilitate their replication. In this study, we demonstrate that PEDV targets Caspase-1 to enhance its replication and suppress IFN-β production. PEDV infection increases the expression of Caspase-1 in both tissues and cells. Overexpression of Caspase-1 significantly reduces IFN-β production while promoting PEDV replication. The suppression of IFN-β production by Caspase-1 is mediated through the cleavage of mitochondrial antiviral signaling (MAVS). Specifically, Caspase-1 cleaves MAVS at Asp182, facilitating viral replication and inhibiting IFN-β production. The resulting MAVS fragments, once cleaved, lose their ability to both inhibit viral replication and induce IFN-β production, thereby enabling PEDV proliferation. Additionally, we observe that Caspase-1 exhibits species-specific cleavage effects on MAVS, though its impact on MAVS cleavage remains consistent. This study provides a novel target for anti-PEDV therapeutic strategies.

## Introduction

Coronaviruses (CoVs) are enveloped, positive-sense, single-stranded RNA viruses capable of infecting a wide range of hosts and exhibiting potential for cross-species transmission. PEDV, first identified in 1971, is characterized by acute, severe watery diarrhea, vomiting, and high mortality, particularly in suckling piglets [1–4]. Viral infection triggers antiviral immune responses [5]. However, viruses are known to disrupt the production of pro-inflammatory cytokines, interferons (IFNs), and IFN-stimulated genes (ISGs) to facilitate their replication [6]. Previous studies have shown that PEDV can restrain the host innate immune response through various strategies. Specifically, PEDV infection downregulates HDAC1, a negative regulator of PEDV replication [7]. The interferon regulatory factor 7 (IRF7), which is essential for type I IFN induction during viral infections, interacts with PEDV M to suppress type I IFN production, thereby promoting PEDV replication [8]. Additionally, PEDV N directly interacts with TBK1, inhibiting TBK1-induced IFN-β production [9]. PEDV also enhances its replication by interacting with various host proteins [10,11]. For instance, PEDV nsp2 targets and promotes the degradation of TBK1 through NBR1-mediated selective autophagy. Notably, the recombinant PEDV lacking nsp2 (rPEDV-Δnsp2) significantly enhances IFN-β and ISGs in vivo, underscoring the pivotal role of nsp2 in subverting the host antiviral response [12]. As research progresses, understanding virus-host interactions will improve prevention and treatment strategies.

Mitochondrial antiviral signaling (MAVS), also known as IPS1, VISA, or CARDIF, plays a crucial role in regulating host innate immune signaling pathways [13–16]. RIG-I-like receptors (RLRs) are crucial RNA-recognition receptors that detect viral RNA and initiate antiviral immune responses [17–20]. RLRs activate MAVS to trigger the activation of the transcription factor IRF3 and induce type I IFNs [21]. MAVS consists of three domains: a N-terminal CARD domain essential for MAVS signaling, a proline-rich central domain, and a C-terminal transmembrane domain (TM) that anchors MAVS to mitochondria [13]. As a key component of the antiviral innate immune response, MAVS is frequently targeted or disrupted by various viruses to promote their replication [22–27].

Caspase-1, the first identified member of the caspase family, is expressed as an inactive zymogen [28]. Upon activation, the protease effector domain of procaspase-1 undergoes cleavage into large and small subunits through auto-processing, forming active complexes required for enzymatic activity [29]. Activated Caspase-1 cleaves pro-inflammatory cytokines including interleukin-1β (IL-1β) and interleukin-18 (IL-18), converting them into their active forms, which are critical for initiating and propagating inflammatory responses [30,31]. Although Caspase-1 is a significant component of the antiviral response, its activation can sometimes create favorable conditions for viral replication under certain circumstances [32]. Excessive Caspase-1 activation and the associated inflammatory response can lead to tissue damage and contribute to chronic inflammatory diseases [33]. Upregulation of Caspase-1 has been observed in various diseases, including in HIV-infected patients, where Caspase-1 levels are dramatically increased post-infection [34].

In this study, we demonstrate that PEDV infection significantly elevates the expression of Caspase-1 in porcine small intestinal tissue and IPEC-J2 cells. Stabilized Caspase-1 not only promotes viral replication but also inhibits the production of IFN-β. Furthermore, Caspase-1 enhances PEDV replication by disrupting IFN-β signaling through its interaction with MAVS. Interestingly, Caspase-1 cleaves MAVS at Asp182, an event that is critically dependent on its enzymatic activity. In contrast to full-MAVS, a mutant MAVS lacking the Caspase-1 cleavage site substantially promotes IFN-β production, both in the presence and absence of Caspase-1. Additionally, the cleavage-site mutant of MAVS exhibits a significantly higher capacity to stimulate IFN-β production. Further investigation of the post-cleavage MAVS fragments revealed that they are unable to inhibit viral replication or stimulate IFN-β production, likely due to a reduced ability to interact with TBK1 and IKK-ε, two key signaling proteins involved in the induction of IFN-β. The oligomerization capacity of the MAVS fragments was notably diminished compared to full-length MAVS. Additionally, human Caspase-1 cleaves MAVS at Asp276, indicating a species-specific cleavage site, although both human and porcine Caspase-1 cleavage events exert similar effects. These findings unveil a previously unrecognized mechanism by which PEDV evades the antiviral innate immune response.

## Materials and methods

### Plasmids and antibodies

The following constructs were previously constructed: S-HA-Caspase-1, H-HA-Caspase-1, S-Myc-MAVS, S-Flag-MAVS, H-Myc-MAVS, and S-HA-Caspase-1-C285A. Various isoforms and mutants of MAVS were generated using the eukaryotic expression plasmids S-Myc-MAVS, S-Flag-MAVS, and H-Myc-MAVS. The primers employed in this study are listed in Table S1a. Antibodies used in this study included anti-Flag, anti-IRF3, and anti-GAPDH (Abclonal Technology); anti-HA (3724), anti-p-IRF3 Ser396, and anti-Caspase-1 (2225) (Cell Signaling Technology); anti-Myc antibody and anti-FLAG magnetic beads (10004D) (Sigma); anti-PEDV N monoclonal antibody (prepared in our laboratory as previously described); HRP-labeled goat anti-rabbit IgG and goat anti-mouse IgG (Hangzhou Fudebio). Transfection reagents Lipo8000™ (Beyotime) and Lipofectamine™ 2000 (Invitrogen) were also used. V×765was obtained from MedChemExpress (MCE), and poly(I:C) was purchased from Merck.

### Cell culture and transfection

IPEC-J2 and HEK293T cells were cultured in Dulbecco’s Modified Eagle’s Medium (DMEM) supplemented with 10% fetal bovine serum (FBS, ExCell) and 1% penicillin-streptomycin (Gibco). These cells were seeded in 24-well or 6-well plates, and plasmid transfection was performed when the cell density reached 60–80%. Transfection was carried out using a transfection reagent according to the manufacturer’s protocol. Additionally, 200 ng/mL of poly(I:C) was introduced into the cells as required. The Caspase-1-specific inhibitor VX-765 (10 µM) was obtained after plasmid transfection.

### Viral infection

The PEDV strain ZJ15XS0101 (GenBank accession no. KX550281) was isolated and stored in our laboratory [35]. PEDV infection was initiated when IPEC-J2 cells reached 80–90% confluence or at a specific time point after plasmid transfection. Various volumes of the virus were used to achieve different multiplicities of infection (MOI). The infected PEDV tissue from piglets naturally infected with PEDV in a pig farm in Zhejiang province and the healthy tissue from previously stored in our laboratory.

### Immunoblotting

Cells were lysed using RIPA lysis buffer (Beyotime Biotechnology) containing 1% phenylmethanesulfonyl fluoride (PMSF, Beyotime Biotechnology) to collect total protein. The proteins were separated using 10% SDS-PAGE gels (Fudebio) and subsequently transferred onto polyvinylidene difluoride (PVDF) membranes (Bio-Rad). The molecular weights of the proteins were determined using a prestained protein marker (180 kDa Plus Prestained Protein Marker, Vazyme Biotechnology, MP201–01) with a range of 12–180 kDa. After blocking with QuickBlock™ Western Blocking Buffer (Beyotime Biotechnology) for several minutes, the PVDF membranes were incubated with the corresponding primary antibodies, followed by incubation with secondary antibodies. Chemiluminescent signals were developed using the ECL Super Kit and analyzed with the Clinx imaging system (Clinx Science Instruments).

### RNA extraction and RT-qPCR

Total RNA was isolated using FreeZol Reagent (Vazyme Biotechnology). A total of 1000 ng RNA was reverse-transcribed into cDNA using HiScript III RT SuperMix for qPCR (+gDNA wiper) (Vazyme Biotechnology). The resulting cDNA served as a template for quantitative PCR analysis using ChamQ Universal SYBR qPCR Master Mix (Vazyme Biotechnology). Primers used for RT-qPCR are listed in Table S1b.

### Co-immunoprecipitation

Cells were lysed with IP lysis buffer (Beyotime Biotechnology) containing 1% PMSF to collect total protein. The cell lysate was centrifuged at 12,000 xg for 10 minutes, and the supernatant was transferred to a new tube. The supernatant was then incubated with anti-Flag binding beads (Sigma A2220) at 4°C overnight. After centrifugation, the beads were washed five times with Co-IP buffer, and 1× loading buffer was added to the eluted proteins. The immunoprecipitated products were subjected to SDS-PAGE and immunoblotting.

### Confocal immunofluorescence assay

HEK293T cells were seeded on coverslips in 24-well plates and transfected with the indicated plasmids for 24 hours. The cells were washed with PBS, fixed with Immunol Staining Fix Solution (Beyotime), and permeabilized with Immunostaining Permeabilization Solution containing saponin (Beyotime). Blocking was performed using QuickBlock Blocking Buffer for Immunol Staining (Beyotime), followed by overnight incubation with primary antibodies at 4°C. After washing three times with PBS, secondary antibodies were incubated for 1 hour at room temperature. DAPI solution was then added to the cells, and incubation continued for 5–10 minutes at room temperature. Images were acquired using a laser confocal microscope (Olympus).

### SDD-AGE

Crude mitochondria were isolated from HEK293T cells, resuspended in sample buffer (0.5× TBE, 10% glycerol, 2% SDS, and 0.0025% bromophenol blue), and loaded onto a vertical 1.5% agarose gel. After electrophoresis in running buffer (1× TBE and 0.1% SDS) for 45 minutes at 100 V and 4°C, immunoblotting was performed.

### Luciferase reporter assay

HEK293T cells were seeded in 24-well plates and co-transfected with pRL-TK and IFN-β. 24 h post-transfection, cells were harvested, lysed in Luciferase Lysate Buffer, and Firefly luciferase activities were measured using a luminometer with the Dual-Luciferase Reporter Gene Assay Kit (Beyotime).

### Statistical analysis

All experiments were performed independently at least three times. Data were presented as the mean ± standard deviation (SD), analyzed and used for statistical graphing by GraphPad Prism 9, the significance of differences was determined by One-way ANOVA with Tukey’s post hoc test or Student’s *t*-test. The significance of differences ranked as: ****stands for *p < 0.0001*, ***stands for *p < 0.001*, ** stands for *p < 0.01*, * stands for *p < 0.05* and ns stands for non-significant difference.

## Results

### Caspase-1 attenuates IFN-β signaling during PEDV infection

**Figure 1.**
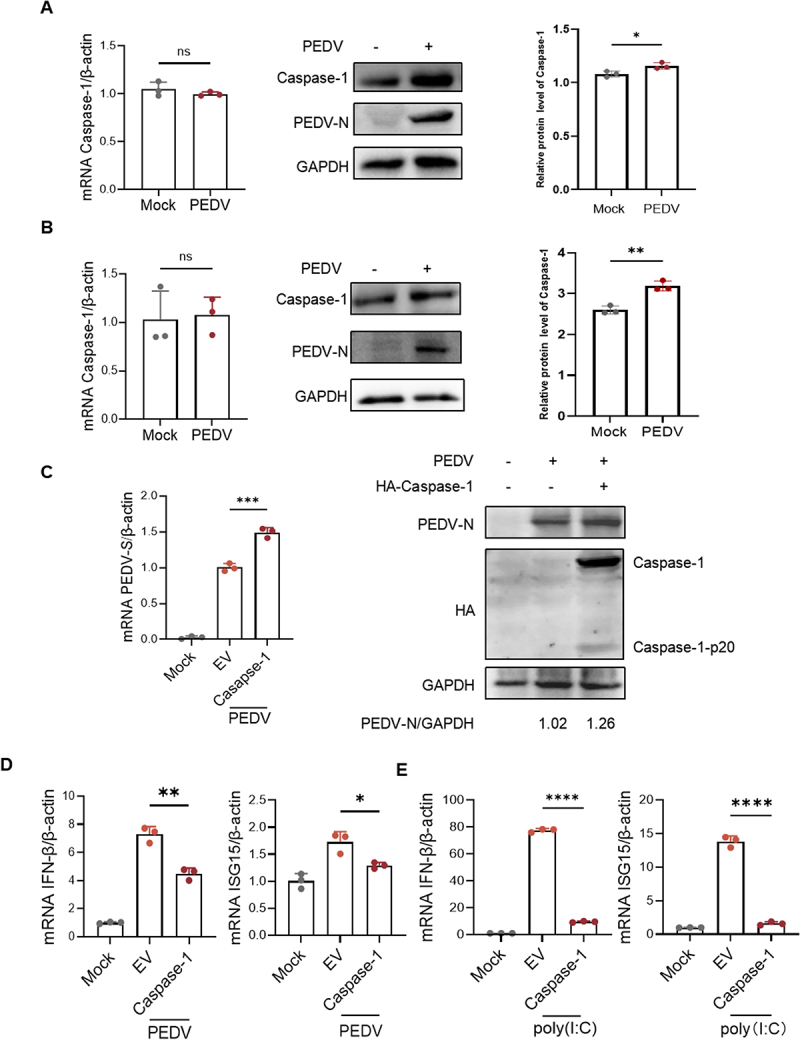
Caspase-1 attenuates IFN-I signaling during PEDV infection. (A) the intestinal tissue samples were collected from healthy or PEDV-infected piglets, RNA was extracted for RT-qPCR, and protein for immunoblotting. Quantitative analysis of relative protein levels obtained according to immunoblotting result. (B) IPEC-J2 cells were non-infected or infected with PEDV at MOI=0.5. After infected 24 h, the cells were collected and subjected to RT-qPCR and immunoblotting. Quantitative analysis of relative protein levels obtained according to immunoblotting result. (C) IPEC-J2 cells were transfected with HA-Caspae-1 or EV (empty vector) for 24 h, following by PEDV infection. At 24 h post-infection (hpi), the cells were collected and subjected to immunoblotting and RT-qPCR. (D) IPEC-J2 cells were transfected with HA-Caspase-1 or EV. After 24 h of transfection, the cells were collected for immunoblotting. (E) IPEC-J2 cells were transfected with HA-Caspase-1 or EV for 24 h, following by poly(I:C) transfection, after 12 h of transfection. RNA was extracted for RT-qPCR. All results shown are representative of at least three independent experiments (ns, *p* > 0.05; *, *p* < 0.05; **, *p* < 0.01; ***, *p* < 0.001; ****, *p* < 0.0001).

Previous studies have shown that PEDV suppresses gasdermin D (GSDMD) levels by cleaving it via Nsp5 [35]. However, the mechanisms through which PEDV may exploit host cell proteins to exacerbate this effect remain unclear. To investigate potential host factors targeted by PEDV, we focused on Caspase-1, a key regulator upstream of GSDMD. Porcine small intestinal tissues were collected from piglets naturally infected with PEDV, and Caspase-1 expression was assessed at both the protein and mRNA level via RT-qPCR and immunoblotting. Notably, PEDV infection resulted in an upregulation of Caspase-1 at the protein level without altering mRNA expression (Figure 1(A)). Similar results were observed in IPEC-J2 cells (Figure 1(B)), prompting further investigation into whether PEDV stabilizes Caspase-1 to facilitate its replication. When IPEC-J2 cells were transfected with HA-tagged Caspase-1 plasmids and subsequently infected with PEDV, viral replication was significantly enhanced (Figure 1(C)). As illustrated in (Figure 1(C)), overexpression of Caspase-1 exhibited a marked activation during PEDV infection. Given the pivotal role of interferon (IFN)-mediated antiviral responses, particularly IFN-β and ISG15, in host defense, we next explored the effect of Caspase-1 on this pathway. As anticipated, Caspase-1 markedly suppressed both PEDV-induced and poly(I:C)-stimulated the production of IFN-β and ISG15 (Figure 1(D, E)). These findings suggest that PEDV promotes replication by stabilizing Caspase-1, which in turn inhibits IFN-β production.

### Caspase-1 targets MAVS to inhibit IFN-β signaling

**Figure 2.**
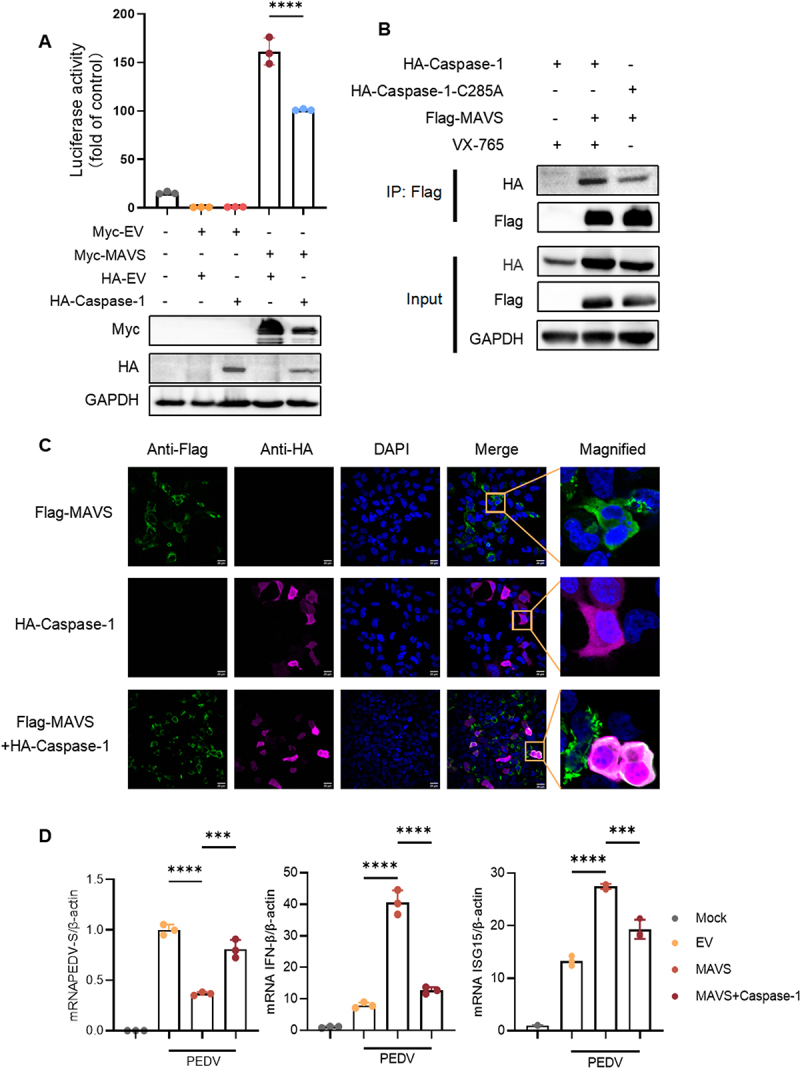
Caspase-1 targets MAVS to inhibit IFN-β. (A) HEK293T were transfected with IFN-β-luc and pRL-TK plasmids, Myc-MAVS or Myc-EV together with HA-EV or HA-Caspase-1. After 24 h of transfection, cells were collected for a dual-luciferase assay and immunoblotting. (B) HEK293T cells were co-transfected with Flag-MAVS and HA-Caspase-1, or Flag-MAVS and HA-Caspase-1-C285A for 24 h. After 4 h of transfection, the cells were treated with VX-765(10 μM). After 24 h of transfection, the cell lysates were subjected to a Co-immunoprecipitation assay with anti-Flag antibody and subsequent immunoblotting. (C) the plasmids of Flag-MAVS and HA-Caspase-1 were respectively or together transfected into HEK293T for 24 h, subcellular localiaztion of Flag-MAVS (red), HA-Caspase-1 (green), and DAPI (blue, nucleus marker) were visualized with confocal microscopy. (D) IPEC-J2 cells were transfected with Myc-MAVS and HA-Caspase-1 for 24 h, the cells were extracted with RNA for RT-qPCR. All results shown are representative of at least three independent experiments (*, *p* < 0.05; ***, *p* < 0.001; ****, *p* < 0.0001).

To better understand how PEDV suppresses IFN-β production through Caspase-1. We focused on MAVS, a critical mediator in the antiviral signaling pathway. MAVS is instrumental in the innate immune response to viral infections. To determine whether Caspase-1 impedes IFN-β production via MAVS, we transfected HEK293T cells with porcine MAVS and Caspase-1 and conducted luciferase reporter assays. The results demonstrated that Caspase-1 inhibited MAVS-mediated IFN-β promoter activation and reduced MAVS expression (Figure 2(A)). Based on these observations, we hypothesized that PEDV might inhibit IFN-β production by promoting the interaction between Caspase-1 and MAVS. Previous research results indicate that activated Caspase-1 cleaves GSDMD to produce N-terminal GSDMD (GSDMD-p30) fragments with perforating activity on cell membrane, which leads to pyroptosis, and it also cleaves pro-IL-1β to produce IL-1β. VX-765 acts by covalent modification of the catalytic cysteine residue in the active site of Caspase-1. Caspase-1 enzyme activity is potently inhibited by VX-765. Given the cytotoxic effects of Caspase-1 overexpression, we implemented VX-765 cotreatment in Co-immunoprecipitation (Co-IP) experiments, a critical optimization that maintained cellular integrity for robust protein interaction profiling. Co-IP experiments confirmed a direct interaction between the two proteins (Figure 2(B)). Interestingly, a Caspase-1 mutant (C285A), which lacks enzymatic activity, still interacted with MAVS (Figure 2(B)). Confocal microscopy further corroborated the co-localization of Caspase-1 and MAVS (Figure 2C). Transfection of IPEC-J2 cells with MAVS and Caspase-1, following by PEDV infection, revealed that while MAVS expression reduced viral replication, Caspase-1 significantly enhanced viral replication and suppressed both IFN-β and ISG15 production (Figure 2(D)). These findings suggest that Caspase-1 interacts with MAVS to inhibit interferon production and promote viral replication.

### Caspase-1 cleaves MAVS at Asp182

**Figure 3.**
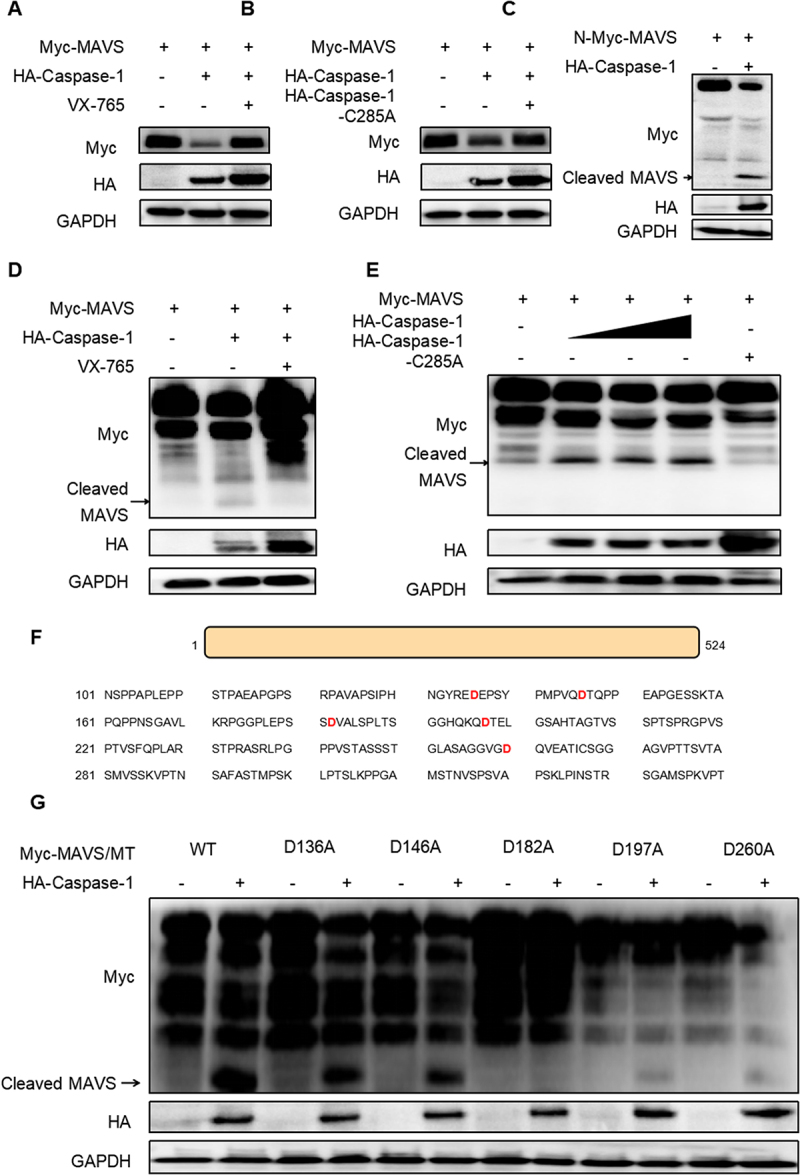
Caspase-1 cleaves at MAVS Asp182. (A) the plasmids of Myc-MAVS and HA-Caspase-1 were transfected into HEK293T cells, and the VX-765 or DMSO were added when the medium was replaced 4 h after transfection. After 24 h transfection, cells were collected for subsequent immunoblotting. (B) HEK293T cells received transfections with the plasmids of Myc-MAVS and HA-Caspase-1,or Myc-MAVS and HA-Caspase-1-C285A for 24 h. Cells were harvested for analysis using immunoblotting. (C) HEK293T cells were transfected with N-Myc-MAVS and with or without HA-Caspase-1 for 24 h, cells were collected for subsequent immunoblotting. (D) the methods same with (A). (E) HEK293T cells were co-transfected with Myc-MAVS and HA-Caspase-1-C285A or HA-Caspase-1 at a different concentrations for 24 h, the cells were processed for immunoblotting. (F) protein sequence of MAVS (101-330aa). Its potential cleavage site is marked in red. (G) HEK293T cells were co-transfected with wild-type (WT) Myc-MAVS or its mutants (D136A/D146A/D182A/197A/D260A) together with EV or HA-Caspase-1. Cells were collected for subsequent immunoblotting. All results shown are representative of at least three independent experiments.

Given that Caspase-1 possesses enzymatic activity, we investigate whether its effect on MAVS is dependent on this activity. HEK293T cells were transfected with MAVS and Caspase-1, followed by treatment with the Caspase-1 inhibitor VX-765. VX-765 effectively rescued MAVS from Caspase-1-induced inhibitor (Figure 3(A)). To confirm these findings, co-transfection of MAVS and Caspase-1, or Caspase-1-C285A, into HEK293T cells yielded consistent findings (Figure 3(B)). We next sought to determine whether Caspase-1 cleaves MAVS through its enzymatic activity. Myc-tagged MAVS plasmid, containing N-terminal Myc tags, was co-transfected with Caspase-1 into HEK293T cells, and subsequent immunoblotting identified cleavage products of approximately 20 kDa (detected by anti-Myc) (Figure 3(C)). Furthermore, VX-765 treatment impaired MAVS cleavage (Figure 3(D)), and Caspase-1-C285A failed to cleave MAVS (Figure 3(E)). These results collectively indicate that Caspase-1 cleaves MAVS through its enzymatic activity. Given the stringent specificity of Caspase-1 for cleaving substrates containing aspartic acid (Asp/D) [36], we examined the possible cleavage sites based on the size of the cleaved bands. In (Figure 3(F)), the Asp residues in the MAVS 101–300 amino acid sequence are marked. To pinpoint the specific cleavage site, HEK293T cells were transfected with Caspase-1 and MAVS, or its mutants (D136A/D146A/D182A/D197A/D260A). The results showed that the D182A mutant could not be cleaved, whereas the other mutants remained cleavable. Therefore, we identified Asp182 as the critical cleavage site (Figure 3(G)).

### Cleavage of MAVS by Caspase-1 inhibits IFN-β production and promotes viral replication

**Figure 4.**
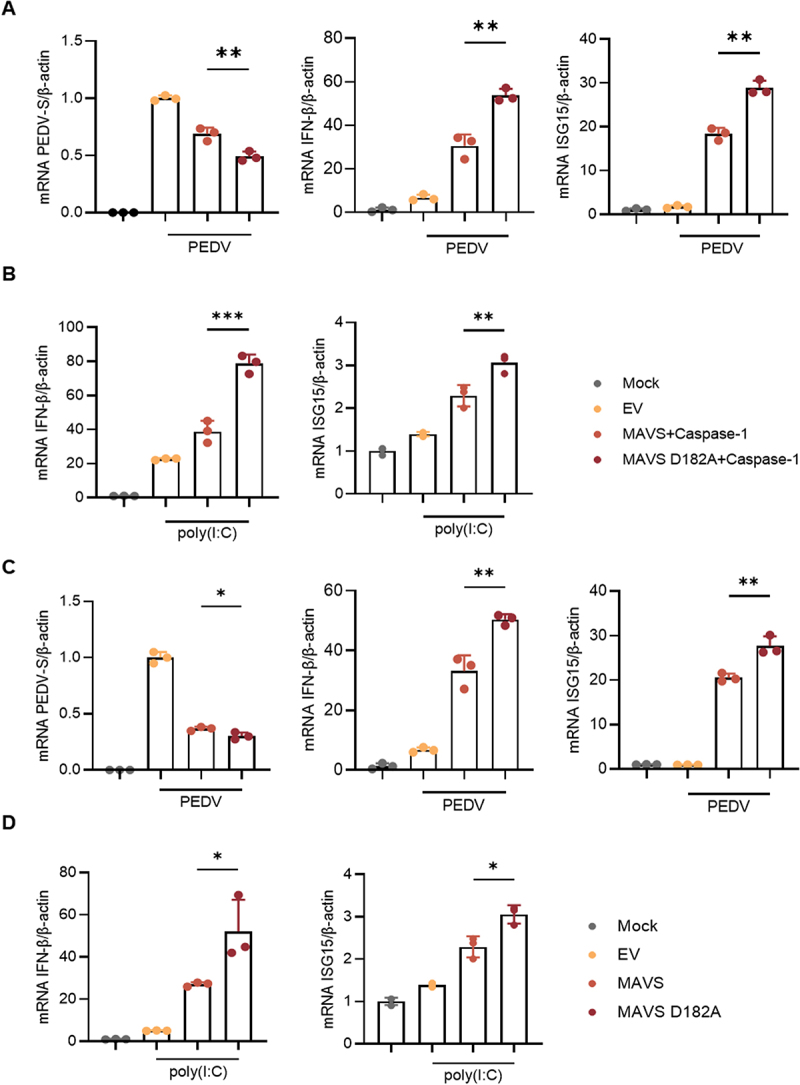
Cleavage of MAVS by Caspase-1 inhibits IFN production to promote viral replication. (A) IPEC-J2 were co-transfected with HA-Caspase-1 and Myc-MAVS or Myc-MAVS-D182A or EV for 24 h, followed by PEDV infection for 24 h at MOI=0.5. The cells were collected for RT-qPCR. (B) IPEC-J2 were co-transfected with HA-Caspase-1 and Myc-MAVS or Myc-MAVS-D182A or EV. After 24 h of transfection, the poly(I:C) was transfected into cells for 12 h. The cells were collected for RT-qPCR. (C) IPEC-J2 were transfected with Myc-MAVS or Myc-MAVS-D182A or EV for 24 h, followed by PEDV infected of 24 h at MOI=0.5. The cells were collected for RT-qPCR. (D) IPEC-J2 were transfected with Myc-MAVS or Myc-MAVS-D182A or EV for 24 h. After 24 h of transfected, the poly(I:C) were transfected into cells for 12 h. The cells were collected for RT-qPCR. All results shown are representative of at least three independent experiments (*, *p* < 0.05; **, *p* < 0.01; ***, *p* < 0.001).

To investigate the effects of MAVS cleavage on viral replication and IFN-β production, IPEC-J2 cells were transfected with Caspase-1 and either MAVS or the MAVS-D182A mutant, followed by PEDV infection. As shown in (Figure 4(A)) a significant reduction in viral replication was observed, accompanied by a marked increase in both IFN-β and ISG15 production. Additionally, upon stimulation with poly(I:C), co-transfection of MAVS-D182A and Caspase-1 in IPEC-J2 cells resulted in a substantial increase in IFN-β and ISG15 production compared to the co-transfection of MAVS with Caspase-1 (Figure 4(B)). Furthermore, when IPEC-J2 cells were transfected with either MAVS or MAVS-D182A and subsequently infected with PEDV or stimulated with poly(I:C), the MAVS-D182A mutant induced superior activation of IFN-β and ISG15 production compared to the MAVS (Figure 4(C, D)). Notably, under PEDV infection conditions, the MAVS-D182A led to a significant reduction in PEDV replication. These findings collectively suggest that Caspase-1 cleavage of MAVS diminishes its antiviral activity, while mutation of the cleavage site enhances its antiviral potential.

### None of the cleaved fragments of MAVS effectively activate IFN-β

**Figure 5.**
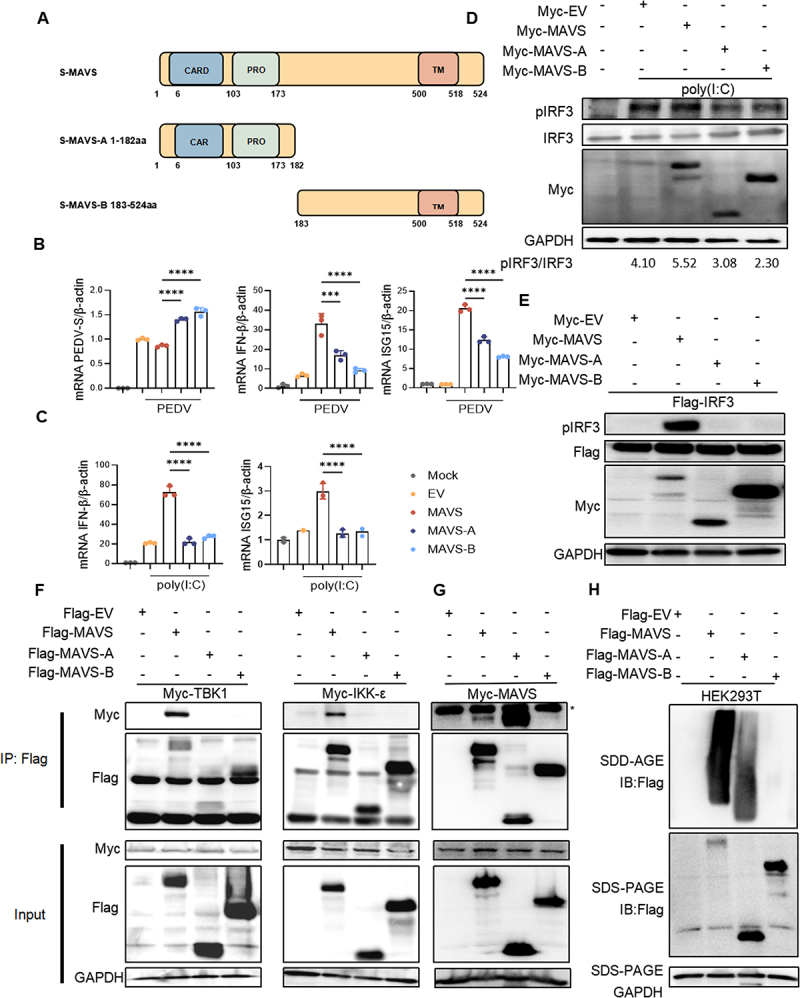
None of the cleaved fragments of MAVS could effectively activate IFN-β. (A) schematic representation of MAVS and its cleaved fragments. (B) IPEC-J2 were co-transfected with Myc-MAVS or Myc-MAVS-A or Myc-MAVS-B or EV for 24 h, followed by PEDV infection for 24 h at MOI=0.5. The cells were collected for RT-qPC. (C and D) IPEC-J2 cells were transfected with Myc-MAVS, Myc-MAVS-A, or Myc-MAVS-B. The cells were transfected poly(I:C) 12 h after transfected plasmids 24 h. Cells were collected for subsequent RT-qPCR and immunoblotting. (E) HEK293T were co-transfected with Flag-IRF3 and Myc-MAVS, Myc-MAVS-A, or Myc-MAVS-B for 24 h. Cells were collected for subsequent immunoblotting. (F) HEK293T cells co-transfected with Myc-TBK1/Flag-IKK-ε and Flag-MAVS or Flag-MAVS-A or Flag-MAVS-B were lysed and immunoprecipitated with anti-Flag beads and analyzed by immunoblotting. (G) the Myc-MAVS and Flag-EV, Flag-MAVS, Flag-MAVS-A or Flag-MAVS-B plasmids were transfected into HEK293T cells, lysed and immunoprecipitated with anti-Flag beads and analyzed by immunoblotting. *, indicated non-specific bands. (H) HEK293T were transfected Flag-EV, Flag-MAVS, Flag-MAVS-A or Flag-MAVS-B for 24 h. The cells were collected and subjected to subcellular fractionation to obtain crude mitochondria. Crude mitochondria were use to examine MAVS aggregation. All results shown are representative of at least three independent experiments (***, *p* < 0.001; ****, *p* < 0.0001).

To evaluate the effect of MAVS cleaved fragments on the innate immune response and viral replication, we generated two truncated forms of MAVS, MAVS-A (1-182aa) and MAVS-B (183-524aa) (Figure 5(A)). IPEC-J2 cells were transfected with MAVS, MAVS-A, or MAVS-B and subsequently infected with PEDV to assess PEDV replication and IFN-β production. The results showed that full-length MAVS significantly induced IFN-β production and suppressed PEDV replication, while both MAVS-A and MAVS-B failed to exhibit similar activity (Figure 5(B)). A similar pattern was observed upon poly(I:C) stimulation following plasmid transfection into IPEC-J2 cells (Figure 5(C)). Under viral infection conditions, MAVS is known to promote polymerization through CARD-CARD interactions, which recruit IRF3 by IKK-ε and TBK1, leading to the activation of IFN-β. To further investigate these interactions, we examined the phosphorylation of IRF3 in response to MAVS cleaved fragments. IPEC-J2 cells were transfected with MAVS, MAVS-A, and MAVS-B, followed by poly(I:C) stimulation for 12 hours. Phosphorylation of endogenous IRF3 was then assessed (Figure 5(D)). Additionally, we evaluated the phosphorylation of exogenous IRF3, revealing that cleaved MAVS fragments were unable to activate IRF3 (Figure 5(E)). We then assessed whether the cleaved MAVS fragments retained the ability to recruit IKK-ε and TBK1, facilitating MAVS polymerization. HEK293T cells were co-transfected with TBK1 or IKK-ε and MAVS, MAVS-A, or MAVS-B demonstrated that only full-length MAVS was capable of binding to TBK1 and IKK-ε (Figure 5(F)). Notably, MAVS-A contains a CARD domain essential for MAVS polymerization, while MAVS-B possesses a TM domain that directs it to the mitochondria. Both the CARD and TM domains of MAVS are indispensable for the induction of IFN-β. To further examine whether cleaved MAVS fragments affect MAVS polymerization. We analyzed their ability to oligimerize. As shown in (Figures 5(G)), MAVS-A was able to associate with MAVS; however, its oligomerization was significantly reduced compared to full-length MAVS. These likely accounts for the inability of the cleaved MAVS fragments to activate IFN-β.

### Species differences in the cleavage of Caspase-1 on MAVS

**Figure 6.**
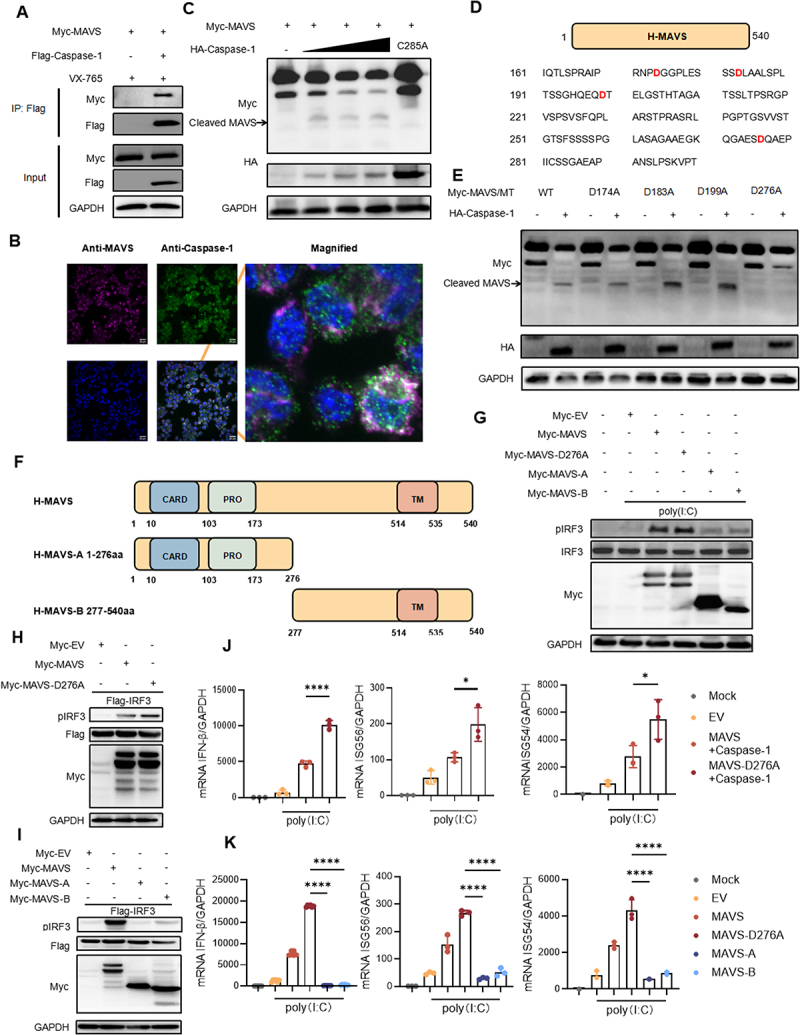
Species differences in the cleavage of Caspase-1 on MAVS. (A) HEK293T cells were co-transfected with Flag-Caspase-1 and Myc-MAVS, and the VX-765 were added when the medium was replaced 4 h after transfection. Cell lysed and immunoprecipitated with anti-Flag beads and analyzed by immunoblotting. (B) subcellular localiaztion of THP-1 MAVS (red), Caspase-1 (green), and DAPI (blue, nucleus marker) were visualized with confocal microscopy. (C) HEK293T cells were co-transfected with Myc-MAVS and HA-Caspase-1-C285A or HA-Caspase-1 at a different concentrations for 24 h, the cells were processed for immunoblotting experiments. (D) protein sequence of MAVS (161-300aa). (E) HEK293T cells were co-transfected with wild-type (WT) Myc-MAVS or its mutants (D174A/D183A/D199A/D276A) together with EV or HA-Caspase-1. Cells were collected for subsequent immunoblotting. (F) schematic representation of MAVS and its cleaved fragments. (G) HEK293T were transfected with Myc-MAVS, Myc-MAVS-A, or Myc-MAVS-B. The cells were transfected poly(I:C) 12 h after 24 h of transfection. Cells were collected for subsequent immunoblotting. (H) HEK293T were co-transfected with Flag-IRF3 and Myc-MAVS, Myc-MAVS-D276A for 24 h. Cells were collected for subsequent immunoblotting. (I) HEK293T were co-transfected with Flag-IRF3 and Myc-MAVS, Myc-MAVS-A, or Myc-MAVS-B for 24 h. Cells were collected for subsequent immunoblotting. (J) HEK293T cells were co-transfected with HA-Caspase-1 and Myc-MAVS or Myc-MAVS-D276A. The cells were transfected poly(I:C) 12 h after 24 h of transfection. RNA were extracted from cells for RT-qPCR. (K) HEK293T cells were transfected with Myc-MAVS, Myc-MAVS-D276A, Myc-MAVS-A, Myc-MAVS-B. The cells were transfected poly(I:C) 12 h after transfected plasmids 24 h. RNA were extracted from cells for RT-qPCR. All results shown are representative of at least three independent experiments (*, *p* < 0.05; ****, *p* < 0.0001).

Next, we explored species-specific differences in Caspase-1 cleavage of MAVS. In a previous analysis, we focused on the cleavage of MAVS by porcine Caspase-1. Here, we extend our investigation to examine the interaction between human-derived Caspase-1 and MAVS. Co-transfection of MAVS and Caspase-1 into HEK293T cells, followed by Co-immunoprecipitation assays, confirmed their interaction (Figure 6(A)). To further validate these findings, we performed immunofluorescence co-localization assays in THP-1 cells, further confirming the endogenous interaction between Caspase-1 and MAVS (Figure 6(B)). We then examined whether Caspase-1 cleaves human MAVS. As shown in (Figure 6(C)), a cleavage band was observed upon co-transfection of MAVS and Caspase-1. These results indicate that MAVS is cleaved by Caspase-1 in both porcine and human species. Based on the substrate recognition properties of Caspase-1, we identified a potential cleavage site within the human MAVS amino acid region 161–300 (Figure 6(D)). As shown in (Figure 6(E)), the cleaved band disappeared when the MAVS-D276A mutant co-transfected with Caspase-1. To further explore these findings, we also constructed truncated forms of MAVS, MAVS-A (1-276aa) and MAVS-B (277-540aa) (Figure 6(F)). We then assessed the phosphorylation of both endogenous and exogenous IRF3. Upon poly(I:C) stimulation, the MAVS-D276A mutant did not affect IRF3 phosphorylation (Figure 6(G, H)). Furthermore, neither of the cleaved MAVS fragments could induce IRF3 phosphorylation (Figure 6(G, I)). To assess the diversity of the ISGs response and the conservation of fundamental antiviral pathways, the effect of human MAVS cleavage on IFN-β and ISGs (ISG54 and ISG56) production were detected. Compared to MAVS and Caspase-1 co-expression in HEK293T cells, the co-expression of MAVS-D276A and Caspase-1 led to enhanced IFN-β production in response to poly(I:C) stimulation (Figure 6(J)). As expected, both MAVS-A and MAVS-B failed to induce IFN-β production (Figure 6(K)). Taken together, these results suggest that MAVS cleavage occurs in both human and porcine cells, and that the cleaved fragments are unable to activate IFN-β production.

**Figure:**
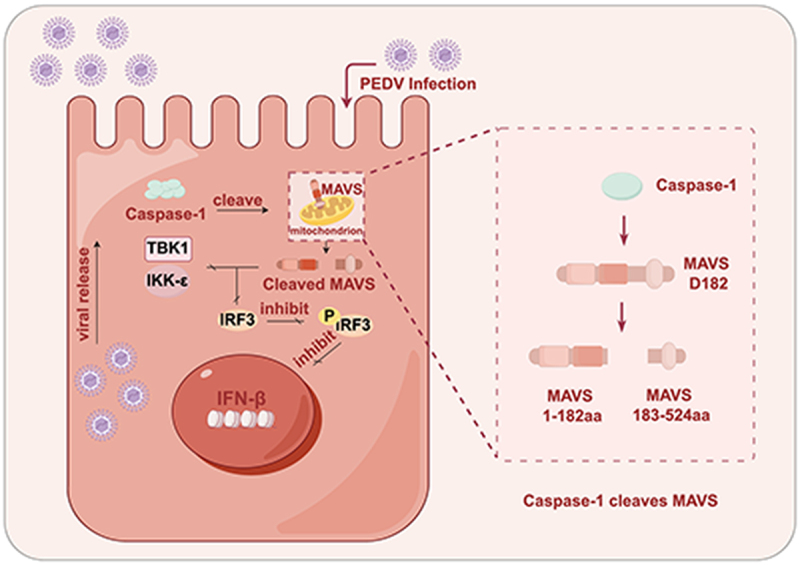

## Discussion

PEDV poses a significant threat to the global pork industry, eliciting widespread concern. Currently, there are no reliable treatments or cures, with current efforts primarily focused on prevention. Despite the development of several vaccine candidates [37–41], substantial limitations remain. Conventional inactivated and attenuated vaccines have proven insufficient in effectively controlling PEDV, highlighting the pressing need for a deeper understanding of the host immune response during PEDV infection. While previous studies have shown that PEDV nsp5 cleaves GSDMD to induce pyroptosis [35], the involvement of host protein pathways in this process remains unclear. In the present study, we demonstrate that the expression of Caspase-1 protein is significantly elevated in PEDV-infected cells compared to uninfected controls, and that Caspase-1 overexpression facilitated PEDV replication.

Our findings further reveal that PEDV inhibits IFN-β production via Caspase-1, thereby promoting its own replication. IFN-β, a critical cytokine in the immune response to viral infections, is targeted by PEDV to evade host defenses. The PEDV M protein interacts with interferon regulatory factor 7 (IRF7), suppressing type I IFN expression and the activation of various antiviral interferon-stimulated genes (ISGs), which in turn facilitates viral replication [8]. Additionally, Zheng et al. demonstrated that the PEDV E protein specifically modulates endoplasmic reticulum stress (ERS) to inhibit type I IFN production [41]. While existing literature primarily attributes PEDV interference with IFN-β production to viral protein, our study uncovers a novel mechanism by which PEDV suppresses IFN-β through the host protein Caspase-1.

Caspase-1 is a key enzyme member of the caspase family, involved in regulating inflammatory responses and processing pro-inflammatory cytokines [29]. Our results shown that Caspase-1 inhibits MAVS-mediated IFN-β production, with evidence of a significant binding between the two proteins. Several studies have suggested that other Caspase family members may also inhibit IFN-β production. For instance, Ning et al. found that activated Caspase-3 cleaves cyclic GMP-AMP synthase (cGAS), MAVS, and IRF3 to prevent excessive cytokine production during viral infections [42]. In this study, we identify that Caspase-1 cleaves MAVS at Asp182, thereby suppressing IFN-β production. MAVS 1-182aa retains the CARD domain (essential for upstream signaling) and proline-rich region, but lacks the transmembrane (TM) domain, disrupting mitochondrial localization and aggregation [43]. MAVS 183-524aa preserves the TM domain (mitochondrial anchoring), yet lacks the CARD domain, abrogating recruitment of RIG-I/IRF3/TRAF complexes [44]. The cleaved MAVS fragments lose their ability to activate IFN-β, as they can no longer bind to downstream signaling proteins. However, another phenomenon has drawn our attention: although the oligomerization of MAVS-A is inferior to full-length MAVS, its binding affinity to full-length MAVS is significantly stronger than that between full-length MAVS. This phenomenon may arise because the oligomerization of full-length MAVS primarily relies on self-aggregation, while the presence of different protein tags may alter its conformation, thereby substantially weakening homotypic binding between full-length MAVS compared to MAVS-A interactions. The precise underlying mechanism warrants further investigation. Interestingly, our findings also showed that human Caspase-1 cleaves MAVS at Asp276, a site distinct from the cleavage site in porcine Caspase-1, suggesting potential species differences.

Remarkably, Caspase-1 plays a central and defining role in the initiation of pyroptosis, a highly inflammatory form of programmed cell death. As the prototypical inflammatory caspase, it functions as the key effector protease downstream of canonical inflammasome activation. Viral infection is a multi-layered interplay between host and pathogen across spatiotemporal dimensions. Following PEDV infection, the viral Nsp5 protein cleaves GSDMD, resulting in its reduction, while the PLP2 protein deubiquitinates and stabilizes Caspase-1 [35,45]. Whether this stabilized Caspase-1 indeed enhances pyroptosis remains an intriguing question. Based on our current results, PEDV infection does not induce LDH release during early stages, attributable to viral protein-mediated inhibition mechanisms. PEDV PLP2 mediates ubiquitin-dependent degradation of the GSDMD-p30 fragment, and PEDV nsp5 cleaves GSDMD into two inactive fragments [35,45]. Notably, pyroptosis intensifies significantly during later PEDV infection. Therefore, we hypothesize that Caspase-1 May initially inhibit IFN-β production, creating conditions for subsequent pyroptosis. Critically, the chronological relationship between IFN suppression and caspase-mediated inflammasome activation and cell pyroptosis requires further rigorous experimental validation.

In our study, we demonstrate that Caspase-1 protein expression increases during PEDV infection. Overexpression of Caspase-1 in IPEC-J2 cells suppresses IFN-β production and promotes viral replication. To investigate how PEDV inhibits IFN-β production, we explored whether Caspase-1 acts on MAVS. Notably, the Caspase-1 inhibitor VX-765 mitigates MAVS reduction caused by Caspase-1. Additionally, we constructed MAVS mutants and identified the specific cleavage site of Caspase-1. Compared to wild-type MAVS, the MAVS-D182A mutant exhibited a significantly enhanced capacity to induce IFN-β production. In contrast, the cleaved MAVS fragments lost their ability to activate IFN-β, likely due to their inability to bind downstream signaling proteins. Remarkably, while previous studies have focused on porcine MAVS, we also examined the Caspase-1-MAVS interaction in humans cells. Although human Caspase-1 cleaves MAVS at a distinct site (Asp276), the overall effect on IFN-β production remains consistent. These findings reveal a previously unrecognized inhibitory pathway through which PEDV modulates IFN signaling, offering valuable insights for the development of novel anti-PEDV therapeutic strategies.

## Conclusion

PEDV has been extensively studied as a major pathogen in swine production. This study reveals that PEDV inhibits IFN-β expression through Caspase-1, thereby promoting its replication. The inhibition occurs via the cleavage of MAVS by Caspase-1, leading to the loss of MAVS ability to activate IFN-β. Furthermore, we demonstrate that this phenomenon is conserved across species. These findings uncover a novel mechanism by which PEDV suppresses IFN-β while enhancing its replication, offering new insights into potential antiviral therapeutic strategies and preventive measures.

## Supplementary Material

Supplementary material.docx

## Data Availability

The data that support the findings of this study are openly available in Figshare at https://doi.org/10.6084/m9.figshare.28573988.v3; The PEDV strain ZJ15XS0101, with the complete genome sequence deposited in GenBank under accession number KX550281.
