# Supplementary material for: Novel strategies for PEDV to interfere with host antiviral immunity through Caspase-1
Source: Virulence. 2025 Sep 13;16(1):2560890. doi: 10.1080/21505594.2025.2560890 (PMC12456220; doi:10.1080/21505594.2025.2560890)
Supplement: Supplementary material.docx [file KVIR_A_2560890_SM7631.docx]

**Supplemental material**

**Table S1a. Primers used in this study for the construction of plasmids.**

| Name | Forward(5’-3’) | Reverse(5’-3’) |
| --- | --- | --- |
| S-Flag-MAVS | CAAGCTTGCGGCCGCGAATTCAATGACGTTTGCCGAGGACA | CCGGGATCCTCTAGAGTCGACTCACTGGGGCAGGCGCCG |
| S-HA-Caspase-1 | GGAGGCCCGAATTCGGTCGACCATGGCCGATAAGGTGCTGA | CATGTCTGGATCCCCGCGGCCGCTTAATGTCCTGGGAAGAGATAAAAAG |
| 1. HA-Caspase-1   -C285A | TTATCCAGGCTGCACGTGGTGAGAAGCAAGGGG | ACGTGCAGCCTGGATAATGATCACCTTGGGTT |
| S-Myc-MAVS  -1-182aa | GGAGGCCCGAATTCGGTCGACTATGACGTTTGCCGAGGACAA | CATGTCTGGATCCCCGCGGCCGCTCAATCAGAGGAGGGCTCCA |
| 1. Myc-MAVS   -183-524aa | GGAGGCCCGAATTCGGTCGACTGTGGCCCTCAGCCCTCT | CATGTCTGGATCCCCGCGGCCGCTCACTGGGGCAGGCGCCG |
| S-Flag-MAVS  -1-182aa | GACGATGACAAGCTTGCGGCCGCAATGACGTTTGCCGAGGACA | CCGGGATCCTCTAGAGTCGACTCAATCAGAGGAGGGCTCCA |
| 1. Flag-MAVS   -183-524aa | AAGGATGACGATGACAAGCTTGTGGCCCTCAGCCCTCTG | CAGGGATGCCACCCGGGATCCTCACTGGGGCAGGCGCCG |
| H-HA-Caspase-1 | CAAGCTTGCGGCCGCGAATTCCATGGCCGACAAGGTCCTG | CCTCTAGAGTCGACTGGTACCTTAATGTCCTGGGAAGAGGTAGAAA |
| 1. Myc-MAVS   -1-276aa | GGGAGGCCCGAATTCGGTCGACAATGCCGTTTGCTGAAGACAA | CATGTCTGGATCCCCGCGGCCGCTCAGTCACTCTCTGCACCCTGTT |
| Myc-MAVS  -277-540aa | GGAGGCCCGAATTCGGTCGACCCAGGCCGAGCCTATCATC | CATGTCTGGATCCCCGCGGCCGCCTAGTGCAGACGCCGCCG |

**Table S1b. Primers used in this study for the RT-qPCR.**

| Name | Forward(5’-3’) | Reverse(5’-3’) |
| --- | --- | --- |
| Porcine  Caspase-1 | CAGGAGTCCTCGAACTCTCCACAG | GGCTCTGAAGACGCAGGCTTAAC |
| Porcine  IFN-β | CCTGGAACAGTTGCCTGGGACT | TCTGCTGGAGCATCTCGTGGAT |
| Porcine  ISG15 | ATGGGTAGGGAACTGAAGGT | CAGACGCTGCTGGAAGG |
| PEDV-S | CGGTTTGTTGGATGCTGTC | AATAAAGAATACGCTGAATGGC |
| Porcine  β-actin | TGCGGCATCCACGAAACTAC | AGGGCCGTGATCTCCTTCTG |
| human  IFN- β | AAACTCATGAGCAGTCTGCA | AGGAGATCTTCAGTTTCGGAGG |
| human  ISG-54 | GCACAGCAATCATGAGTGAGAC | CTGGCCCCTGCAGTCTTTTA |
| human  ISG-56 | TGGCAGCCTAAAGGAGAGGA | CCAGAAATCGGCCATAGTGGA |
| human  GAPDH | GCCTTCCGTGTCCCCACTG | CGCCTGCTTCACCACCTTC |
